# Host PrP Glycosylation: A Major Factor Determining the Outcome of Prion Infection 

**DOI:** 10.1371/journal.pbio.0060100

**Published:** 2008-04-15

**Authors:** Nadia L Tuzi, Enrico Cancellotti, Herbert Baybutt, Lorraine Blackford, Barry Bradford, Chris Plinston, Anne Coghill, Patricia Hart, Pedro Piccardo, Rona M Barron, Jean C Manson

**Affiliations:** 1 Neuropathogenesis Unit, Roslin Institute, Edinburgh, United Kingdom; 2 Center for Biologics Evaluation and Research, Food and Drug Administration, Rockville, Maryland, United States of America; The Scripps Research Institute, United States of America

## Abstract

The expression of the prion protein (PrP) is essential for transmissible spongiform encephalopathy (TSE) or prion diseases to occur, but the underlying mechanism of infection remains unresolved. To address the hypothesis that glycosylation of host PrP is a major factor influencing TSE infection, we have inoculated gene-targeted transgenic mice that have restricted N-linked glycosylation of PrP with three TSE strains. We have uniquely demonstrated that mice expressing only unglycosylated PrP can sustain a TSE infection, despite altered cellular location of the host PrP. Moreover we have shown that brain material from mice infected with TSE that have only unglycosylated PrP^Sc^ is capable of transmitting infection to wild-type mice, demonstrating that glycosylation of PrP is not essential for establishing infection within a host or for transmitting TSE infectivity to a new host. We have further dissected the requirement of each glycosylation site and have shown that different TSE strains have dramatically different requirements for each of the glycosylation sites of host PrP, and moreover, we have shown that the host PrP has a major role in determining the glycosylation state of de novo generated PrP^Sc^.

## Author Summary

In prion infection, disease requires the presence of the endogenous host-encoded prion protein, PrP. PrP is a glycoprotein (modified by the addition of sugar molecules) with two consensus sites for sugars to attach. Different PrP forms are usually observed: one diglycosylated, two different monoglycosylated, and one unglycosylated. How PrP glycosylation influences prion infection remains obscure. We have used three different murine transgenic models, developed with the gene-replacement technique, to investigate each glycotype of PrP contribution to prion diseases, or transmissible spongiform encephalopathies (TSEs). For this purpose, mice expressing mono- or unglycosylated PrP were challenged with different prion strains. Remarkably, we found that glycosylation of host PrP is not mandatory for TSE infection, because mice expressing only unglycosylated PrP were susceptible to infection and able to transmit the disease to other animals. However, we also show that host PrP glycosylation can modulate the infectious process, since strains differ in their ability to infect hosts with restricted PrP glycosylation. These results elucidate the role of glycosylation in prion infection and in particular demonstrate that strains need sugars at specific sites of host PrP to successfully induce prion disease.

## Abstract 3

The primary determinant of the prion infectious process is the glycosylation status of the host prion protein, PrP.

## Introduction

Transmissible spongiform encephalopathies (TSEs), or prion diseases, are fatal neurodegenerative diseases that include scrapie in sheep, bovine spongiform encephalopathy (BSE) in cattle, chronic wasting disease (CWD) in deer and elk and Creutzfeldt-Jakob disease (CJD) in humans. The host-encoded glycophosphatidylinositol anchored prion protein, PrP^C^, is essential for the development of TSE disease [1], and a common feature of these diseases is the accumulation of PrP^Sc^ in the brain, an abnormal form of PrP^C^, which displays partial resistance to proteinase K (PK) digestion. This accumulation is considered to arise via conversion of PrP^C^ to PrP^Sc^ [2], and PrP^Sc^ has been proposed to be the infectious agent. If this is indeed the case, it is not clear how a protein alone can encipher TSE strain information resulting in different incubation times and targeting of pathological lesions in the brain, but it has been suggested that differences in glycosylation and/or conformation [3–6] of PrP could account for these different properties.

PrP has two potential sites for N-linked glycosylation, which are variably occupied, producing di-, mono-, and unglycosylated PrP [7]. The diversity in glycosylation, combined with the complexity of added sugars, results in a large number of glycosylated forms of PrP [8]. These different glycoforms of host PrP have been proposed as a mechanism to determine the targeting of TSE strains to specific brain regions [6]. Moreover, PrP glycoform analysis in the infected host (ratios of di- and monoglycosylated PrP^Sc^, as well as the relative mobility of unglycosylated PrP^Sc^) is increasingly used as a means of distinguishing between TSE strains [9,10] and for identifying and classifying newly emerging strains [11,12]. However, the significance of different glycosylated forms of PrP in the normal function of PrP as well as the disease process is not understood. To investigate the involvement of host PrP glycosylation on TSE disease susceptibility and pathology following infection with different TSE strains, we have used our gene-targeted transgenic mice with restricted host PrP glycosylation and inoculated them intracerebrally with three TSE strains.

Three inbred lines of gene-targeted mice, carrying mutations at the first, second, or both PrP N-linked glycosylation sites—referred to as G1 (N180T), G2 (N196T), and G3 (N180T-N196T) mouse lines, respectively [13]—were used in these experiments. The gene targeting approach ensures that the altered PrP allele remains in the correct genomic location under control of the endogenous promoter [14]. Importantly, this approach allows each line of mice to be compared, and thus the effect of the loss of each glycosylation site on disease pathogenesis can be analysed directly.

Western blot analysis has demonstrated that both G1 and G2 lines lack diglycosylated, but express mono- and unglycosylated PrP, whereas G3 mice only express unglycosylated PrP [13]. PrP in the mutant mice has been shown to have a similar cellular localisation in G1 and G2 mice to that of wild type, whereas G3 mice, which lack all PrP N-linked carbohydrates, have been demonstrated to have mainly intracellular PrP [13]. Despite differences in intracellular localisation, mono- and unglycosylated PrP levels in the brain are similar to those in wild type [13]. Here we establish, using these unique lines of mice, that although host PrP glycosylation is not essential to support TSE disease, it can have a profound effect on capacity of TSE strains to infect the host. Furthermore we demonstrate in vivo that glycosylation of PrP^Sc^ is not essential for TSE strain to transmit infectivity.

## Results

Three homozygous lines (G1/G1, G2/G2, and G3/G3, subsequently referred to as G1, G2, and G3 respectively), together with 129/Ola wild-type mice (which acted as controls, because the gene-targeted mice are inbred lines on a 129/Ola background), were infected intracerebrally (i.c.) with the TSE strains 79A and ME7, which are classified as low and medium glycosylated strains, respectively, according to their glycoform analysis based upon the ratio of the diglycosyl and monoglycosyl bands by Western blotting [15]. Each of these lines of mice was shown to respond in strikingly different ways to infection with each TSE strain.

### G3 Mice Expressing Unglycosylated PrP Differ in Their Response to Various TSE Strains

**Figure 1:**
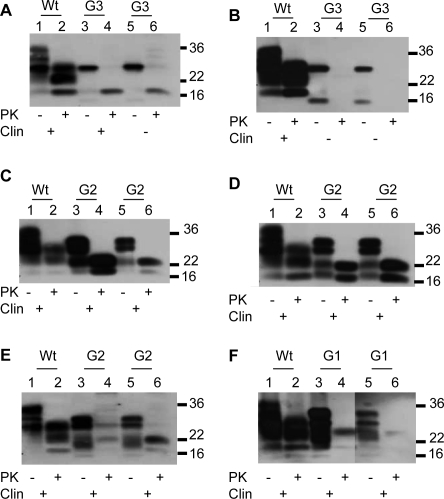
Western Blot Analysis of Brains from TSE Inoculated Glycosylation Mutant Mice Brain homogenates were treated with or without PK as indicated prior to SDS PAGE analysis. (A) G3 and wild-type animals inoculated with 79A. Lanes 1–2: 129/Ola wild-type mice clinically (clin) positive/pathologically (path) positive; lanes 3–4: G3 mice clin positive/path positive; lanes 5–6: G3 mice clin negative/path positive. (B) G3 and wild-type animals inoculated with ME7. Lanes 1–2: 129/Ola mice clin positive/path positive; lanes 3–4: G3 mice clin negative/path negative with no plaques; lanes 5–6: G3 mice clin negative/path negative with plaques. (C) G2 and wild-type animals inoculated with ME7. Lanes 1–2: 129/Ola; lanes 3–6: G2 mice, brains positive for plaques. All animals clin positive/path positive. (D) G2 and wild-type animals inoculated with 79A. Lanes 1–2: 129/Ola mice; lanes 3–6: G2 mice. All animals clin positive/path positive. (E) G2 and wild-type animals inoculated with 301C. Lanes 1–2: 129/Ola mice; lanes 3–6: G2 mice brains positive for plaques. All animals clin positive/path positive. (F) G1 and wild-type animals inoculated with 79A. Lanes 1–2: 129/Ola mice; lanes 3–6: G1 mice. All animals clin positive/path positive. Lanes 1–4: 3-min exposure; lanes 5–6: 10-min exposure. Molecular weight markers indicated in KDa. Wild-type (Wt) mice are 129/Ola mice. Monoclonal antibody 7A12 (kindly provided by M.-S. Sy) was used for immunodetection of PrP.

**Figure 2:**
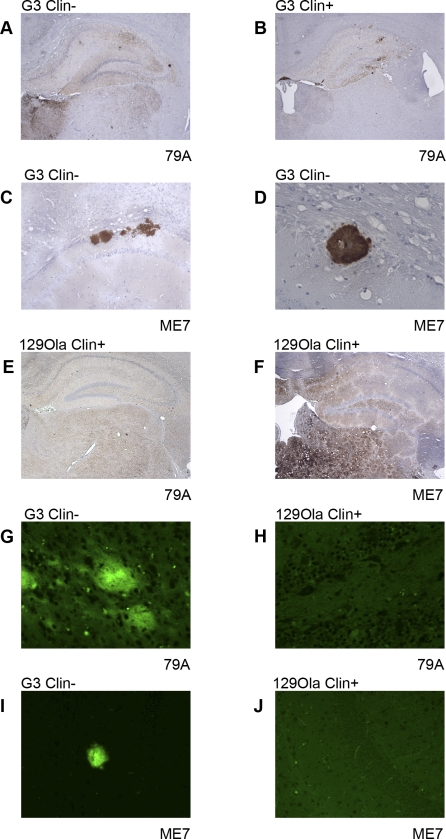
Immunohistochemical Analysis of Brain from G3 Mice Inoculated with TSE Strains 79A or ME7 Sections from the cerebrum and cerebellum were immunostained for PrP with monoclonal antibody 6H4 and analysed by light microscopy using a Nikon Eclipse E 800 microscope. Brain sections obtained from (A) clinically negative, or (B) clinically positive G3 mice after inoculation with 79A showing widespread accumulation of PrP in the hippocampus and thalamus are shown. (C and D) Brain sections from clinically negative G3 mice inoculated with ME7 showing large PrP positive plaques in subcallosal areas. (E and F) Brain section of clinically positive 129/Ola mice infected with 79A (E) or ME7 (F), showing widespread PrP deposition in the hippocampus and thalamus. (G) Thioflavin-S fluorescent PrP-amyloid plaques in the subcallosal region of a clinically negative G3 mouse inoculated with 79A. (H) Thioflavin-S treatment of sections from a clinically positive 129/Ola mouse infected with 79A with no presence of plaques. (I) Thioflavin-S fluorescent PrP-amyloid plaques in a clinically negative G3 mouse infected with ME7. (J) Thioflavin-S staining of a clinically positive 129/Ola mouse infected with ME7 with no presence of plaques. Magnifications: (A), (B), (E), and (F): 4×, (C), (G), (H), and (I): 10×; and (D): 40× .

G3 mice were susceptible to 79A infection, with clinical TSE and spongiform degeneration of the brain being observed in 4/21 animals (Table 1). However, although only four mice presented with clinical TSE disease, with considerably longer and wider range of incubation times (435 ± 92 d) compared with wild-type mice (148 ± 2.6 d), Western blot analysis of six brains all demonstrated PK-resistant PrP, whether or not clinical disease was observed (Figure 1A). Furthermore, immunohistochemical analysis of brains revealed variation in the amount of PrP immunopositivity amongst cases with some animals without clinical signs showing widespread accumulation of PrP in the cerebrum (Figure 2A and 2B), suggesting that a subclinical disease was also present in the G3 animals. Thus, 79A can infect transgenic mice in the absence of glycosylated host PrP, but this lack of glycosylation dramatically lengthens the incubation time of disease suggesting that glycosylation of host PrP facilitates infection by 79A.

**Table 1:**
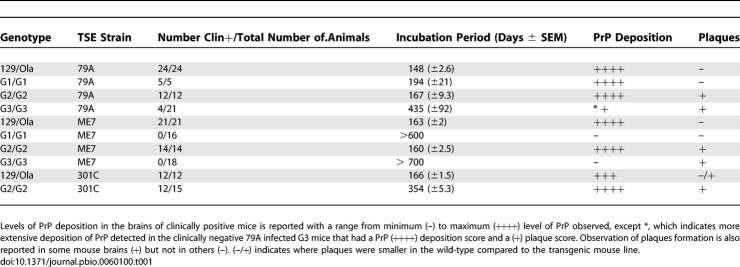
Incubation Periods of Wild-Type and Glycosylation Mutant Mice Infected with TSE Strains

To establish whether the brains of G3 79A-inoculated mice carried infectivity, brain homogenates from one clinically positive (incubation period 456 d) and one subclinical G3 mouse (incubation period 722 d; i.e. clinically negative but positive by immunohistochemistry and showing spongiform degeneration of the brain) were used to inoculate wild-type mice i.c.. For comparison, brain homogenate from a 79A inoculated wild-type (129/Ola) control mouse was also used to i.c. inoculate another group of wild-type mice. The brain extracts from the two 79A/G3 mice and the one 79A/129/Ola mouse were found to contain TSE infectivity (Table 2). The clinically positive 79A/G3 brain homogenate resulted in an incubation period of 197 d (±7.6), whereas the clinically negative 79A/G3 brain generated a shorter incubation time (164 ± 2.2 d), but both G3 brain homogenates produced slightly longer incubation periods that that resulting from 79A/129/Ola (137 ± 1.1 d). Western blot analysis of recipient mouse brains revealed the presence of di-, mono-, and unglycosylated PrP^Sc^ despite the absence of di- and monoglycosylated PrP^Sc^ in 79A inoculated G3 mouse brain (unpublished data). These data indicate that glycosylated PrP^Sc^ is not a prerequisite for the transmission of infectivity.

**Table 2:**
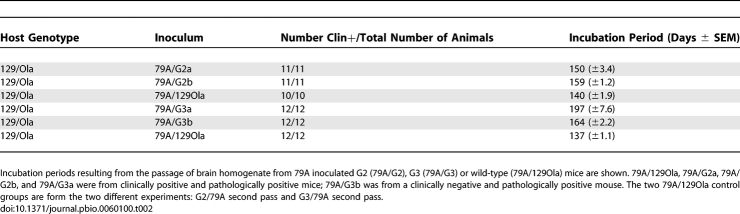
Second Passage of G2 and G3 Mouse Brain Inoculated with 79A; Incubation Periods of 129/Ola Mice

In contrast, G3 mice inoculated with ME7 did not show clinical signs of TSE disease or spongiform degeneration in the brain as determined by lesion profiling and were thus scored as pathologically negative [16]. Brains from selected animals analysed by Western blotting showed no PK-resistant PrP (Figure 1B) up to 740 d post inoculation (Table 1). Despite the lack of these indicators of TSE disease, brains of a number of animals showed PrP-positive plaques in the vicinity of the corpus callosum and, in one case, adjacent to the lateral ventricle; diffuse PrP deposits were not detected (Figure 2C and 2D). Thioflavin-S–treated brain sections confirmed the presence of PrP amyloid plaques in 79A- (Figure 2G) and ME7-inoculated G3 mice (Figure 2I). To determine whether the presence of the PrP amyloid plaques was in response to TSE inoculation rather than an innate ability of G3 mice to form plaques, age-matched G3 mice (24 animals in total) that were injected with brain homogenates from wild-type controls were examined but found to have no PrP accumulation in the brain (unpublished data). In addition, in agreement with previous studies [13], PrP accumulation was not observed in older (600 d) uninjected G3 mice. Therefore, the PrP accumulation in the brain of the mice inoculated with 79A and ME7 was clearly in response to inoculation with a TSE agent rather than due to any effect of the mutation in host PrP per se. Whether this PrP accumulation is associated with infectivity requires further subpassage of G3/ME7 brains; however, tissue was not available from this experiment to carry out this subpassage and therefore will be examined in future studies.

### G2 Mice Differ in Their Response to Scrapie or BSE-Derived Strains

**Figure 3:**
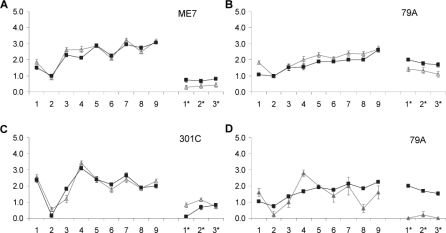
Lesion Profile Analysis of TSE-Inoculated PrP Glycosylation Mutant Mice G2 and 129/Ola mice were inoculated i.c. with TSE strains ME7 (A), 79A (B), or 301C (C) and G1 mice were inoculated i.c. with 79A (D). Squares: wild type; open triangles: G2; and solid triangles: G1. All mice (12 in each group) used in these analyses were clinically and pathologically positive. Nine gray matter areas: 1, dorsal medulla; 2, cerebellar cortex; 3, superior colliculus; 4, hypothalamus; 5, medial thalamus; 6, hippocampus; 7, septum; 8, cerebral cortex; 9, forebrain cortex and three white matter areas: 1*, cerebellar white matter; 2*, mesencephalic tegmentum; 3*, pyramidal tract (*x*-axis) were scored on scales of 0–5 for gray and 0–3 for white matter areas (*y*-axis). The mean scores for each area are shown (error bars ± SEM). No differences between wild-type and G2 mice were observed after inoculation with ME7 (A) or 79A (B); however some differences in the superior colliculus and white matter areas were observed between the two groups when inoculated with 301C (C). Differences were observed between wild-type and G1 mice inoculated with 79A (D) especially in area: 2, 4, 8, and in the white matter.

**Figure 4:**
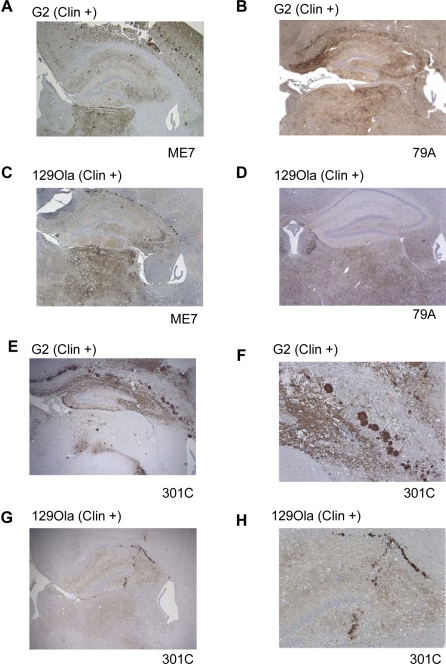
Immunohistochemical Analysis of Brain from G2 Mice Inoculated with TSE Strains ME7, 79A, and 301C Brain sections were immunostained for PrP using monoclonal antibody 6H4 and analysed by light microscopy using a Nikon Eclipse E 800 microscope. Brains from clinically positive G2 mice inoculated with (A) ME7 or (B) 79A showing wide spread PrP deposition. Brains from 129/Ola wild-type mice inoculated with (C) ME7 or (D) 79A also showing wide spread PrP deposition. The TSE strain 301C was used to inoculate (E and F) G2 mice, which showed wide spread plaque-like and fine punctuate PrP deposits in the cerebrum and cerebellum or (G and H) 129Ola mice, which also showed plaque-like PrP deposition. All 301C inoculated brains shown were from clinically positive mice. Magnifications: (A–E) and (G), 4×; (F) and (H), 10×.

In contrast to G3 mice, all G2 mice were found to develop clinical signs of disease when inoculated with ME7 and presented with similar incubation periods to wild-type controls (160 ± 2.5 d and 163 ±2 d, respectively) (Table 1). Spongiform degeneration, determined by lesion profile analysis, of G2 mouse brains was comparable to wild-type mice both in the distribution and degree of vacuolation (Figure 3A). Wide spread, fine punctuate, coarse, and plaque-like PrP immunopositive deposits were detected in ME7-infected animals (Figure 4A). Mono- and unglycosylated PrP^Sc^ was detected by Western blot analysis in brains of ME7-inoculated G2 mice, whereas di-, mono- and unglycosylated PrP^Sc^ was detectable in wild-type, inoculated mice (Figure 1C).

**Figure 5:**
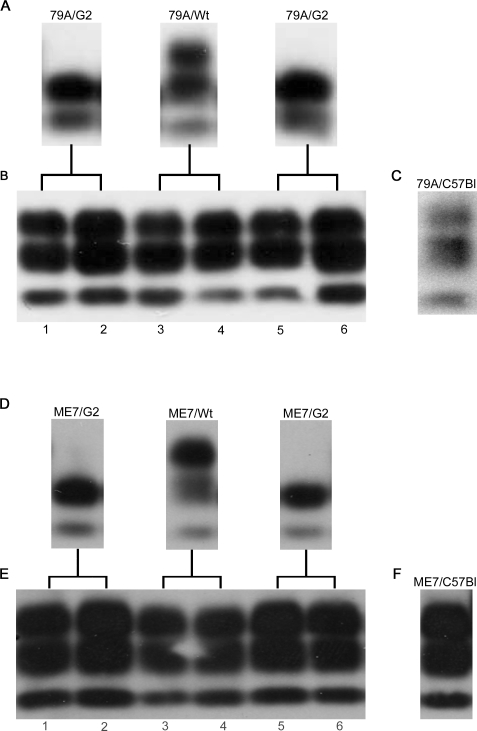
Western Blot Analysis of Brains from Second Passage of 79A-Infected G2 and Wild-Type Mice Brain homogenates from (A) clinically and pathologically positive 79A inoculated G2 or wild-type, 129/Ola (Wt), mice were used to i.c. inoculate 129/Ola mice. (B) The 79A inoculated G2 mouse brains lacked diglycosylated PrP^Sc^, but all 129/Ola mice inoculated with 79A/G2 or 79A/Wt brain material had di-, mono-, and unglycosylated PrP^Sc^. (C) Brain of a C57Bl mouse infected with 79A used as comparison for the glycotype. Brain homogenates from (D) clinically and pathologically positive ME7 inoculated-G2 or wild-type, 129/Ola (Wt), mice were used to intracerebrally inoculate 129/Ola mice. (E) The ME7 inoculated G2 mouse brains lacked diglycosylated PrP^Sc^ but all 129/Ola mice inoculated with ME7/G2 or ME7/Wt brain material had di-, mono-, and unglycosylated PrP^Sc^. (F) Brain of a C57Bl mouse infected with ME7 used as comparison for the glycotype. Lanes 1–6: TSE-inoculated 129/Ola mouse brain. All samples were PK treated.

G2 mice were also shown to be susceptible to TSE disease when inoculated with 79A, but whereas the incubation time with ME7 was similar to that in wild-type mice, with 79A, incubation time was significantly prolonged (167 ± 9.3 d for G2/79A and 148 ± 2.6 d for 79A/129/Ola) (Table 1). Spongiform degeneration of G2 mouse brains was also found to be comparable to wild-type mice (Figure 3B). Immunohistochemical analysis of selected 79A-inoculated G2 mouse brains revealed variable amounts of fine punctuate and plaque-like PrP depositions in the cerebrum (Figure 4B). By Western blot analysis, mono- and unglycosylated PrP^Sc^ was detected in brains of 79A-inoculated G2 mice, whereas di-, mono-, and unglycosylated PrP^Sc^ was detectable in wild-type, inoculated mice (Figure 1D). To determine whether the brains of 79A-inoculated G2 mice had TSE infectivity, despite the lack of diglycosylated PrP^Sc^, wild-type mice were inoculated with brain homogenate from 79A-inoculated G2 and wild-type control mice. All mice were found to be susceptible to TSE disease with two independent 79A/G2 brain homogenates producing incubation times of 150 d (±3.4) and 159 d (±1.2) compared to 79A/129/Ola brain homogenate resulting in a slightly shorter incubation period of 140 d (±1.9) (Table 2). The pattern and degree of spongiform degeneration in all subpassage cases, as determined by lesion profile analysis [16], were found to be comparable (unpublished data). Western blot analysis revealed the glycosylation pattern of the PrP^Sc^ in the recipient mouse brain to possess di-, mono-, and unglycosylated PrP^Sc^ (Figure 5B) despite the inoculum possessing only mono- and unglycosylated PrP^Sc^ (Figure 5A).

Moreover, the glycosylation pattern of the original Wt/79A inoculum was not changed after passage through G2 mice. The comparison of the electrophoretic mobility of the 79A in wild-type animals before and after passage through G2 mice revealed the same glycotype with a predominant monoglycosylated band (Figure 5C).

To investigate this effect with another strain of agent, subpassage was performed from clinical positive G2/ME7 brains into wild-type animals. Western blot analysis of recipient wild-type mice also showed fully glycosylated PrP^Sc^ in the brain of the recipient mice, despite the inoculum having only mono- and unglycosylated PrP^Sc^ (Figure 5D and 5E). Furthermore, similarly to the 79A strain, the original Wt/ME7 glycosylation pattern was maintained after passage in G2 mice (Figure 5F). Therefore, it is clear that the ability to generate diglycosylated PrP^Sc^ in response to TSE infection does not depend on the presence of the diglycosylated species of PrP^Sc^ in the infecting inoculum.

We originally chose the 79A and ME7 strains of TSE for use in this study, because glycoform ratio analysis had revealed them to have a different predominance of mono- and diglycosylated PrP^Sc^; with 79A predominantly monoglycosylated, whereas ME7 was more equally di- and monoglycosylated in wild-type mice [4]. Since the disease profiles observed in G2 mice that were infected with ME7 and 79A were similar to those observed in wild-type mice, with the exception of slightly prolonged incubation times in G2/79A mice, we decided to analyse the effect of the second glycosylation site of PrP with a further TSE strain having a different glycoform ratio. For this purpose we inoculated G2 mice and wild-type mice intracerebrally with the BSE mouse-adapted TSE strain 301C, which has a predominance of diglycosylated PrP^Sc^ [15]. In contrast to ME7 and 79A, a very prolonged incubation time was observed in the 301C inoculated G2 mice (354 ± 5.3 d) compared to wild-type mice (166 ± 1.5 d). However, despite the prolonged incubation time in the 301C-inoculated G2 mice, the overall pattern of spongiform degeneration was similar to that observed in wild-type mice (Figure 3C). Analysis by immunohistochemistry showed wide spread, plaque-like, and fine punctuate PrP deposits in G2 mice in the cerebrum and cerebellum (Figure 4E and 4F), and mono- and unglycosylated PrP^Sc^ was detected by Western blot (Figure 1E). These experiments clearly show that this BSE-derived strain does not require diglycosylated host PrP (the predominant characteristic of the glycoform used to identify BSE strains) to be pathogenic; however, the presence of sugars at N196 of host PrP would appear to allow more efficient progression of TSE disease, because the time taken to reach terminal disease is dramatically shorter in wild-type mice having normal PrP^C^ glycosylation. The importance of the presence of glycans at N196 for efficient TSE disease progression is therefore clearly TSE strain dependent.

### G1 Mice Are Susceptible to 79A, but Are Resistant to ME7, Strain Infection

**Figure 6:**
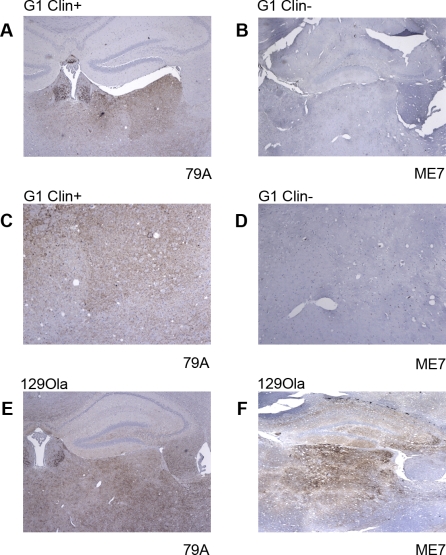
Immunohistochemical Analysis of Brain from G1 Mice Inoculated with TSE Strains 79A and ME7 Brain sections were immunostained for PrP using monoclonal antibody 6H4 and analysed by light microscopy using a Nikon Eclipse E 800 microscope. Widespread PrP immunopositivity is seen mostly in the thalamus (A and C) of clinically positive, symptomatic, G1 mice inoculated with 79A, absence of PrP-immunopositivity in the hippocampus (B) and thalamus (D) of clinically (Clin) negative, asymptomatic, G1 mice inoculated with ME7, (E) widespread PrP deposition in the thalamus and hippocampus of a wild-type mouse (129/Ola) after inoculation with 79A; (F) widespread PrP deposition in the thalamus of a wild-type mouse with neurological signs after inoculation with ME7. Magnifications: (A), (B), (E), and (F), 4×; (C) and (D), 10×.

Having established that the absence of glycans at the second glycosylation site of host PrP can influence PrP deposition and incubation period, albeit in a strain-dependent manner, we next investigated the importance of the first glycosylation site of PrP. This was achieved by inoculating G1 transgenic mice (that lack the ability to attach carbohydrates to the first glycosylation site of PrP; i.e., N180) with 79A or ME7 TSE strains. G1 mice had an extended incubation time when infected with 79A (+ 46 d) compared to wild-type mice (Table 1) and a prominent difference in distribution and degree of vacuolar pathology (Figure 3D). Brains of terminally ill, 79A-infected G1 mice showed mono- and unglycosylated PrP^Sc^ compared to di-, mono-, and unglycosylated PrP^Sc^ present in control 129/Ola mice (Figure 1F). Immunohistochemical analysis revealed widespread PrP deposition in both G1/79A and Wt/79A brain (Figure 6A, 6C, and 6E). However, in striking contrast to infection with 79A, G1 mice inoculated with ME7 appeared resistant to infection, remaining healthy up to 600 days post-inoculation, whereas wild-type controls had an incubation period of 163 ± 2.0 d (Table 1). No PrP^Sc^ was found by Western blot analysis in G1 brains inoculated with ME7 (unpublished data). Moreover, pathologic studies did not show PrP deposition or spongiform degeneration in brains of G1 mice inoculated with ME7 (Figure 6B and 6D) in sharp contrast to wild-type mice that showed wide spread PrP accumulation in the brain (Figure 6F). These results again indicate that TSE strains differ dramatically in their requirements for host PrP glycosylation.

## Discussion

We have demonstrated in vivo that glycosylation of host PrP is not necessary to establish a TSE. Mice expressing only unglycosylated PrP (G3 mice) are capable of supporting a TSE, albeit in a strain-dependant manner, but disease susceptibility is reduced in response to the lack of glycans on host PrP. This differs from a previous study that reported that transgenic mice expressing unglycosylated PrP were resistant to two TSE strains [3]. There are, however, considerable differences between these two studies that may account for the discrepancy including: (i) the expression of hamster rather than mouse PrP genes, (ii) different TSE strains, and (iii) the method of transgenic mouse production. Gene targeting was used to generate the mice described in this study, thus the altered PrP gene is in the normal chromosomal location and under the PrP gene (*Prnp*) control elements. Whereas a previous study by De Armond et al. used transgenic mice generated by random integration of the PrP transgene [3], which can result in disruption of genes at the site of integration, ectopic expression due to the influence of surrounding control elements, as well as high levels of transgene expression, all of which may affect the outcome of disease [14]. Indeed PrP glycosylation mutant transgenic lines produced by random integration and expressing the same transgene have been reported to display different incubation periods with the same TSE strain, preventing direct comparisons between different lines [17].

Our glycosylation-deficient PrP transgenic mice have both an alteration in amino acid sequence and glycosylation. Mutation of either asparagine or threonine in the NXT glycosylation consensus site of murine PrP will prevent the attachment of glycans to asparagine. We altered asparagines at both consensus sites to threonine, and this is regarded as a conservative amino acid alteration. The PrP expressed in our G1 and G2 transgenic lines is localised primarily at the cell surface in a manner comparable to that in wild-type mice and displays no detectable PrP^Sc^-like properties as determined by PK sensitivity, cleavability by phosphatidylinositol-specific phospholipase C (PIPLC), and detergent solubility assays [13]. Moreover, remarkable similarity in the course of infection in parts of this study argues against the substituted amino acids affecting the course of disease but rather that the differences observed can be attributed to differences in glycosylation.

The observation that G3 mice are capable of supporting TSE disease is surprising given that the cellular location of the unglycosylated PrP appears primarily intracellular [13]. This suggests that TSE disease can occur with perhaps complete absence or at least very low levels of PrP on the cell surface. 79A may be able to infect the cell via a mechanism independent of cell surface expression of host PrP^C^, suggesting that transit of normal PrP to the surface of the cell [17,18] may not be an absolute requirement for the development of TSE disease. PrP^Sc^ accumulation in the TSE infected animal is considered to occur through the interaction of PrP^Sc^ with host PrP^C^, resulting in conversion of PrP^C^ to PrP^Sc^. We have established that glycosylation of host PrP is not a requirement for the formation of de novo–generated PrP^Sc^ in vivo, which is in agreement with in vitro studies [19,20]. However, there is still uncertainty as to where PrP^C^ to PrP^Sc^ conversion occurs, with differing reports that it takes place either at the cell surface, extracellularly, in microsomes, along endocytic pathways, or in the cytosol [21–25]. In our studies, the primarily intracellular location of the unglycosylated PrP in G3 mice suggests that either the very low levels of cell surface PrP^C^ expression is adequate to allow the conversion process to occur, or that conversion does not occur at the cell surface; in vitro studies will be necessary to resolve where conversion actually occurs in the G3 mice.

We have shown for the first time, to our knowledge, that glycosylation of PrP^Sc^ is not necessary for the transmission of TSE infectivity to wild-type mice. This study also revealed that the de novo PrP^Sc^ generated in the recipient animals consisted of di-, mono-, and unglycosylated PrP^Sc^, whereas the donor PrP^Sc^ was fully unglycosylated. This is perhaps surprising, considering it is postulated that the donor PrP^Sc^ acts as a template for its amplification [26], yet our results suggest that the host dictates the glyscoylation status of the de novo–generated PrP^Sc^, rather than that of the donor as it has also been suggested in some in vitro experiments [27,28]. The effect of host on PrP^Sc^ glycosylation should therefore be considered when glycoform analysis, which determines the ratio of di- and monoglycosylated PrP^Sc^, as well as the relative mobility of unglycosylated PrP^Sc^, is used to identify TSE strains [29–31] and classify newly emerging ones [12,32].

In contrast to the results obtained using 79A, we found that ME7 was unable to cause clinical TSE disease in mice expressing only unglycosylated PrP^C^. G1 mice (which have glycans only at N196) appear also resistant to TSE disease following ME7 inoculation, whereas G2 mice are fully susceptible to TSE disease with this strain. The fact that the cellular location of PrP^C^ in the G1 and G2 mice are comparable to each other and to wild-type mice [13] indicates that the inability of ME7 to cause TSE disease in the G1 mice is not due to the cellular location of PrP^C^ but rather due to the absence of glycans at N180. Therefore, the inability of ME7 to cause clinical TSE disease in G3 mice may also be due to lack of carbohydrates at N180, rather than the low levels of PrP^C^ expressed at the cell surface.

G1 mice were found to be fully susceptible to TSE disease following 79A inoculation, but with a prolonged incubation time compared to wild-type mice. Similarly, the absence of glycans at N196 resulted in a prolongation of incubation period in 79A-inoculated G2 mice. With the cellular location of PrP in G1 and G2 mice being comparable to that in wild-type mice [13], perhaps the prolongation of incubation period is due to the lack of glycans at N180 or N196, respectively, but whether this is due to a reduced efficiency of uptake, inefficient interaction of the infectious agent with PrP^C^ due to the lack of glycans at either site, or a slightly reduced efficiency of the disease process once the infectious agent has gained access to cells can not be determined from these studies. Experiments are underway to address this issue. In contrast to the observation that the lack of glycans at N196 has little or no effect on TSE disease resulting from inoculation with 79A or ME7, not all G2 mice inoculated with the TSE strain 301C showed clinical signs of TSE disease, and furthermore, those that did had a dramatic extension of the incubation period. However, clinical TSE disease was observed in the majority of 301C-inoculated mice, despite the increase in incubation period, which indicates that this strain does not have an absolute requirement for glycans at N196 but that the presence of them does allow for a more efficient infection.

Despite differences in susceptibility of G2 mice to infection with three different strains, the vacuolation profile of these mice and wild-type mice was similar, suggesting that glycosylation at the G2 site did not influence the strain targeting. Contrastingly, the 79A vacuolation profile in G1 was different when compared to wild type, indicating that glycosylation at the G1 site may have an effect on strain targeting. This finding will be further investigated in future experiments.

Clinical negative G3 mice infected with 79A or ME7 have shown the presence of PrP amyloid plaques in the brain. The formation of amyloid plaques is commonly associated with the pathological process of TSE disease as well as other neurodegenerative diseases [33]. However an alternative view is that plaque formation may actually be a mechanism that the host uses to attempt to protect itself from the potentially harmful effects of oligomers [34]. If this is the case in the TSE diseases, then the lack of clinical disease in the majority of the 79A-infected G3 mice (despite the presence of TSE infectivity as demonstrated by the transmission of TSE disease to wild-type mice) could indicate that the host is attempting to protect itself from the de novo generated abnormal PrP by sequestering it as amyloid. Interestingly, PrP-amyloid plaques have been detected previously in a transgenic mouse model expressing mutant PrP in the absence of clinical signs, spongiform degeneration, and infectivity [35]. Thus a possible explanation for presence of plaques in clinical negative mice may be that amyloid formation can somehow block the propagation of disease within the brain. Our glycosylation mutant transgenic mice may therefore provide a model to investigate the involvement of PrP glycosylation on plaque formation in response to different TSE strains.

In conclusion, we have demonstrated that glycosylation of host PrP is not essential for the host to be susceptible to TSE disease. Moreover, glycosylation of PrP^Sc^ is not necessary for the transmission of TSE infectivity to a new host, and the glycotype of the host PrP has a major influence on the de novo generated PrP^Sc^. Finally, we have shown that host PrP has a major role to play in TSE disease susceptibility and incubation period and that TSE strains differ dramatically in their requirements for host PrP glycosylation in order to allow TSE disease to occur.

## Materials and Methods

### Transgenic mouse lines.

Inbred gene targeted transgenic mouse lines G1, G2, and G3, and the corresponding inbred 129/Ola wild-type control line have been described previously [13]. All gene-targeted transgenic mice used in this study were homozygous.

### Genotyping of mouse tail DNA.

A portion of tail was removed post-mortem from each mouse. DNA was prepared from a 1-cm piece of tail by digestion overnight at 37 °C in tail lysis buffer (300 mM sodium acetate, 1% SDS, 10 mM Tris pH 8, 1 mM EDTA, 200 μg/ml PK) and subsequent extraction with an equal volume of phenol/chloroform. DNA was precipitated with isopropanol, washed with 70% ethanol, and re-suspended in 100 μl of TE buffer (10 mM Tris, 1 mM EDTA pH 7.4). The mismatch PCR method to identify the different transgenics has been described elsewhere [13].

### Preparation of inoculum and injection.

Inocula were prepared from the brains of C57BL mice with terminal ME7, 79A, or 301C TSE disease. A 1% homogenate of each sample was prepared in sterile saline prior to use as an inoculum. All mice were genotyped by PCR, grouped, and coded prior to intracerebral inoculation with 20 μl of inoculum under anaesthesia. For second passage experiments, separate inocula were prepared from the brains of two G2/79A infected mice with terminal TSE disease as well as from the brain of one 129/Ola (wild-type) mouse with terminal 79A scrapie as control. A 1% homogenate of each sample was prepared in sterile saline prior to use as an inoculum for inoculation of 129/Ola wild-type mice. All experimental protocols were submitted to the Local Ethical Review Committee for approval before mice were inoculated. All experiments were performed under license and in accordance with the UK Home Office Regulations (Animals (Scientific Procedures) Act 1986).

### Scoring of clinical TSE disease.

The presence of clinical TSE disease was assessed as described previously [16]. Animals were scored for clinical disease without reference to the genotype of the mouse. Genotypes were confirmed for each animal by PCR analysis of tail DNA at the end of the experiment. Incubation times were calculated as the interval between inoculation and cull due to terminal TSE disease. Mice were killed by cervical dislocation at the terminal stage of disease, at termination of the experiment (between 500–700 d), or for welfare reasons due to intercurrent illness.

### Lesion profiles.

Sections were haematoxylin and eosin stained and scored for vacuolar degeneration on a scale of 0 to 5 in nine standard gray matter areas and a scale of 0 to 3 in three standard white matter areas as described previously [16].

### Immunohistochemistry.

Sections were stained for disease associated PrP using monoclonal antibody 6H4 (1/10000; Prionics). Antigen retrieval by autoclaving at 121 °C for 15 min, and 5 min formic acid (98%) treatment was used to facilitate the detection of PrP. Sections were then blocked with normal rabbit serum prior to incubation with the primary antibody. Antibody binding was detected with the Catalyzed Signal Amplification System (Dakocytomation) and visualized with diaminobenzidine (DAB). In all the experiments, normal brain homogenate-infected and PrP-null negative controls were used. Images were taken using a Nikon Eclipse E800 microscope. Sections were analysed by an observer blinded to the mouse genotype and type of inoculum.

### Thioflavin-S treatment.

Brain sections were stained with haematoxylin for 30 s and then washed in water. Sections were treated with a thioflavin-s solution (10 mg/ml) for 5 min and then with a 70% ethanol solution for 5 min. After washes with water, sections were mounted using Fluorescent Mounting Medium (Dakocytomation) and analysed using a Nikon Eclipse E400 microscope with a FITC filter.

### Western blotting.

Mice were killed by cervical dislocation and brains and spleens were removed, flash frozen in liquid nitrogen, and then stored at −70 °C until required. Half or whole brains were weighed and mechanically homogenized from frozen in nine volumes of ice cold NP40 lysis buffer (1% Nonidet 40, 0.5% sodium deoxycholate, 150 mM NaCl, 50 mM Tris pH 7.5), with the addition of phenylmethysufonyl fluoride (PMSF) (final concentration 1μM; Sigma) to prevent protein degradation by endogenous proteases. The homogenate was centrifuged at 8000 revolutions per minute for 10 min to remove unhomogenised debris. Total protein was denatured in 1× Novex Tris-Glycine SDS Sample Buffer (Invitrogen Life Technologies) and 1× NuPage Sample Reducing Agent (Invitrogen Life Technologies) for 30 min at 95 °C. Proteins were separated by gel electrophoresis at 125 V using Novex Pre-cast Tris-Glycine gel (12% or 14% acrylamide, Trisglycine; Invitrogen Life Technologies). Proteins in the acrylamide gel were transferred to polyvinylindene fluoride (PVDF) membrane at 25 V (125 A/gel) using a semi-dry transfer blotter (Bio-Rad) in 1X Transfer Solution (48 mM Tris, 39 mM Glycine, 0.375 % sodium dodecyl sulphate (SDS), 20% methanol).

## Abbreviations

BSE: bovine spongiform encephalopathy
i.c.: intracerebrally
PK: proteinase K
PrP: prion protein
TSE: transmissible spongiform encephalopathy
